# Questioned Virginity Has No Definite Reply

**DOI:** 10.1007/s10508-022-02332-5

**Published:** 2022-04-12

**Authors:** Abeer Ahmed Zayed, Reham Nafad Elbendary, Asmaa Mohammad Moawad

**Affiliations:** Department of Forensic Medicine and Clinical Toxicology, Faculty of Medicine, Cairo University, Kasr Alainy Street, Cairo, 11562 Egypt

**Keywords:** Virginity tests, Egypt, Sexual assault, Hymen

## Abstract

Virginity is the nullity of sexual experience in females. However, the use of virginity testing as proof of previous involvement in sexual relations is dependent on having an intact hymen, which refers to a hymen with no signs of previous penetrating injury. Although the quality of this evidence in questioned virginity is extremely poor and considered a violation of human rights, it still constitutes a major facet in medicolegal investigations of sexual assaults. This work evaluates virginity testing as currently practiced in many countries, including Egypt, in terms of medical and legal considerations.

## Introduction

Virginity is defined as the state of abstinence from sexual intercourse (Grarmaroudi et al., 2010). In most conservative cultures, especially Arab communities, the state of female virginity is linked with chastity, morality, and purity (Abboud et al., 2015). It also reflects on family honor. Thus, determining virginity is an important social issue in many countries, such as Afghanistan, Egypt, Iran, Jordan, Palestine, South Africa, Turkey, and Uganda (Ferentinos, 2008; Olson & García-Moreno, 2017). In addition, increasing reports of virginity testing in some communities have emerged in countries like the Canada, Netherlands, Spain, and Sweden (Independent Forensic Expert Group, 2015).

In practice, testing virginity relies on the presence of an intact hymen, a remnant of tissue at the opening to the vagina that remains after the solid vagina “hollows out” during embryological development (Schlafer & Foster, 2016). This test considers an intact hymen as equivalent to virginity based on the hypothesis that sexual intercourse is the only cause of a torn hymen. However, this concept is no longer logically or medically accepted (Mishori et al., 2019) due to the test’s subjectivity, which outweighs the average physician’s reliability, techniques, and experience, making conclusions beyond their skills (Anwer et al., 2017).

As practiced in many countries, the test is commonly performed on unmarried females, mostly without their consent, or on individuals who are unable to give consent—detainees, women alleging rape, or women accused of prostitution by authorities—and as part of controlling sexuality through social and public policies (Independent Forensic Expert Group, 2015).

In 2016, a member in the Egyptian parliament called for virginity tests for university entry as a way to combat premarital sex (Fenton, 2016). In Egypt, these examinations are performed by gynecologists, nurses, and midwives to prove to the family that a girl has not had premarital relations (Zeyneloğlu et al., 2013). In Indonesia, such a test is required for military job applications by unmarried women (Smith & Newey, 2019). Despite being officially banned in Afghanistan, it is still practiced for official and unofficial reasons on a family’s request (Nader & Mashal, 2017). In some instances, the test is carried at the request of a female because of what is called “virginity fraud” reflecting the deep-rooted fears and concerns about the issue, especially before marriage. These fears and thoughts would negatively affect proper engagement in healthy marital relations between newly married couples. However, in most cases, it is carried out for the benefit of third parties to prove that someone is a virgin, which brings with it a sense of powerlessness, humiliation, worthlessness, and lack of the right to self-determination for the woman subjected to it (Crosby et al., 2020).

Given this context, in October 2018, to eradicate violence practiced against women and girls, the World Health Organization (WHO), United Nations Human Rights Office, and UN Women stated that virginity testing was nonscientific and a violation of human rights. They concluded that “this medically unnecessary, and often painful, humiliating and traumatic practice must end” (Crosby et al., 2020).

The case presented below is a model of the subjectivity at hand in virginity testing. It embodies the fallacies surrounding any conclusive opinion derived from the test or when considered by individuals or legal practitioners as proof of virginity. This case was a turning point in our career as forensic doctors and opens many questions and considerations regarding its usefulness.

## Case Report

An 18-year-old non-married female came, with her mother, to the forensic department of Kasr Alainy hospitals, seeking a certificate of virginity. Kasr Al Ainy Hospital is a 3000-bed facility in the metropolitan area of Cairo and is the largest tertiary referral center in Egypt and the Middle East region (El Dib, 2015). The mother claimed that her daughter’s conduct had been questioned by her family because of her illegal relationship with one of their neighbors. She stated that the daughter was examined by a gynecologist, who said that the hymen showed signs of previous injury but did not issue a written report. The girl admitted sexual contact with her friend but denied penile penetration. After confirming the mother and daughter’s identities, consent for examination was taken from the latter and signed by the former.

The examination was performed by a direct inspection with intense illumination, while the girl was lying in the lithotomy position and knee-chest position. The hymen was inspected both at rest and while being borne down upon. Indirect stretch of the hymen edge was used to verify the hymen’s features. The examiners were two forensic consultants, who were confused because of the dilated hymen opening, the redundant edges, and thickened consistency. As a result, their decision was not conclusive. Another senior consultant concluded that the hymen was intact. So, there were multiple inspections by at least three examiners, none of which served a medical purpose, only a social one.

## Discussion

According to the WHO, sexuality is “a core part of being human throughout life that involves sex, gender identities and roles, sexual orientation, eroticism, pleasure, intimacy, and reproduction.” It manifests itself in beliefs, attitudes, values, behaviors, practices, roles, and relationships and is impacted by a complex interplay of biological, psychological, social, religious, spiritual, cultural, legal, and historical variables (World Health Organization, 2006).

Premarital sexual activity among African adolescents and adults is a critical issue in sexuality studies (Olamijuwon & Odimegwu, 2022). Virginity is a symbol of pride, dignity, and respect in many African countries, including Egypt, and motivates girls to be wanted and pursued. A woman’s virginity is considered necessary to attract “a good man” (Bhana, 2016). The value of virginity and chastity is also promoted through a variety of national, cultural, and religious programs that, left unchecked, might have far more negative implications than benefits for young girls and women. More crucially, despite clear evidence that it has no scientific basis, the routine use of hymenal blood or other forms of virginity testing may put women at risk of negative experiences (World Health Organization, 2014).

The acceptance of hymen examination as an indicator of virginity is denied by many researchers, as the hymen shows a great range of variation such that we cannot nominate specific features regarding the shape and width of the opening, thickness, elasticity, or the position within the vagina (Mishori et al., 2019). The natural opening of an intact hymen may be annular, cribriform, crescentic, or septate. The edges of the opening may be regular and stretched or redundant and folded, giving the appearance of a dentate and fimbriate hymen commonly misdiagnosed as ruptured. The thickness of the hymen is greatly variable between the thin, fragile mucous membrane and thick, fleshy, and flexible tissue that allows nontraumatic penetration (Berenson, 1995; Curtis & San Lazaro, 1999; Mishori et al., 2019). At puberty, estrogenization of the hymen occurs with consequent thickening, increases in width and changes in the position from deeply situated to a few centimeters inside the vagina (Brämswig & Dübbers, 2009).

The morphology of this membrane may be altered due to causes other than sexual activity, such as accidental penetrative trauma, insertion of foreign objects or fingers, and surgical procedures (Berkowitz, 2011; Goodyear-Smith & Laidlaw, 1998a, 1998b). Moreover, confusion may occur where normal variations are mistaken for signs of sexual trauma or abuse, as in cases of genital erythema and nevi, anal fissures, hymenal opening enlargement, edge narrowing, clefts, partial notching of the hymen, and failure of its midline fusion (Hillard, 2013). Accordingly, a girl may be falsely labeled sexually experienced and thus are at risk for various adverse consequences, ranging from social stigmatization to honor killing (Hegazy & Al-Rukban, 2012).

On the other hand, sexually experienced women may be declared virgins. Sexually active women may have no peculiar abnormal hymen features, due to its elasticity (Olson & García-Moreno, 2017). At the same time, the hymen has an amazing ability to repair itself after injuries, especially in young females (Sawyer Sommers, 2007). Adding to the complexity of the issue, there has been an increase in hymen reconstruction surgery, such as hymenorrhaphy or hymenoplasty, to repair deflorated hymens and cause vaginal bleeding on the next occasion of intercourse as proof of virginity (Cook & Dickens, 2009; Goodyear-Smith & Laidlaw, 1998a).

Another crucial consideration concerns the exact constituents of sexual intercourse. Confusion about them leads to a questioned state of virginity in the case of a girl who has not had vaginal intercourse but engaged in other forms of sexual connections. According to academic and media accounts from American sources, many young people are substituting nonvaginal sexual practices for vaginal intercourse like oral or anal sex in order to keep “technical virginity” in order to “remain pure” or avoid “sinful” activities (Uecker et al., 2008).

Medical evidence from previous studies supports our position that hymenal physical examination is an unreliable indicator of prior sexual intercourse (Anderst et al., 2009; Mishori et al. 2019; Olson & García-Moreno, 2017; Pillai, 2008). One study reported that 52% of cases of women who admitted having past intercourse still had intact hymens (Adams et al., 2004).

In Turkey, a survey of forensic physicians revealed that about two-thirds of the genital findings of virginity examinations conducted twice on the same females were contradictory, where 73% of the contradictory findings were made by general practitioners or gynecologists (Anderst et al., 2009).

In an Iranian study, Robatjazi et al. (2016) examined the experiences of 16 physicians and midwives who performed virginity tests. The clinicians admitted that the test was unreliable in determining virginity.

In the USA, Kellogg et al. (2004) studied 36 pregnant adolescents who reported sexual abuse. Nonspecific genital-examination findings were observed in 22 (64%) cases, inconclusive findings in 8 (22%) cases, suggestive findings in 4 (8%) cases and 2 (6%) cases had findings of definite evidence of vaginal penetration. Another study in the USA compared the genital features of 192 girls with vaginal-penetration history from sexual abuse with 200 who denied past penetration. Among both groups, only 2.5% of positive findings were unique to the group with vaginal penetration history (Berenson et al., 2002).

The same confusion as in our case occurred in Turkey, where a 14-year-old sexually abused girl was subjected to hymenal examination three times because the court was confused by different expert opinions, from one forensic expert and two gynecologists (Faikoglu et al., 2007).

In Egypt, a study of 200 participants regarding virginity tests found that 82% of unmarried and 95% of the married participants refused premarital tests, while 65% of unmarried and 82% of married females did not know whether the law prohibited forced virginity tests (Gaber & Rasoul, 2019).

Egyptian law defines rape as the slightest penetration of the labia of a living female by a male organ, with or without hymen defloration (Fulu & Miedema, 2015). This legal framework views virginity more broadly than the hymen’s physical state.

According to Muslim law, the required proof of sexual contact in the act of *zinā* is four eyewitness testimonies to the actual act of penetration or confessions repeated four times and not retracted (Quran, surat Alnour verse 2). The Maliki legal school also considers an unmarried woman’s pregnancy to be evidence of *zinā* which means that the hymen’s state is not an issue according to Muslim law.

### Conclusion and Recommendations

Through our research on reproductive and sexual issues, we conclude that virginity is not an anatomical feature. We recommend the complete ban of the practice of virginity testing in different countries. However, this test is both a culturally mediated and complex practice that is considered a violation of human rights by major international groups. Nonetheless, this practice plays an important role in marriage and the social status of women. Changing social beliefs, attitudes, and practices like virginity testing, female genital mutilation, and child marriage must come from within the culture.

Religious and community leaders’ engagement is crucial for making such a change. Education and knowledge about the unreliability of virginity testing and its possible adverse effects should be provided to communities through training their members and providing them with reading materials on reproductive health.
